# The functional roles of competitive endogenous RNA (ceRNA) networks in apoptosis in human cancers: The circRNA/miRNA/mRNA regulatory axis and cell signaling pathways

**DOI:** 10.1016/j.heliyon.2024.e37089

**Published:** 2024-08-31

**Authors:** Mina Shahpari, MohamadReza Hashemi, Tayebeh Younesirad, Aida Hasanzadeh, Mohammad mahdi Mosanne, Mohamadreza Ahmadifard

**Affiliations:** Department of Medical Genetics, School of Medicine, Babol University of Medical Sciences, Babol, Iran

**Keywords:** CeRNA network, CircRNA, MiRNA sponge, Apoptosis, Signaling pathway, Cancer

## Abstract

Circular RNAs are noncoding RNAs with circular conformation mainly due to backsplicing event. CircRNAs can potentially impact cell biological processes by interacting with cell signaling pathways. Numerous circRNAs have been found to be aberrantly expressed in a variety of cancers. These RNAs can act as ceRNA (competitive endogenous RNA) by sponging certain miRNAs to form circRNA/miRNA/mRNA networks. Dysregulation of ceRNA networks may lead to dysfunctions in various cell pathways, which modulate apoptosis-associated genes and ultimately result in cancer progression.

Since disruption of apoptosis is one of the leading causes of cancer development, one approach for cancer treatment is to drive cells toward apoptosis.

In this review, we present a summary of studies on the role of ceRNA networks in cellular signaling pathways that regulate apoptosis; these networks are suggested to be potential biomarkers for cancer treatment.

## Abbreviations

ceRNA: Competitive endogenous RNA
miRNA: MicroRNA
circRNA: Circular RNA
ncRNA: Non-coding RNA
MREs: miRNA response elements
Apaf-1: Apoptotic protease–activating factor-1
TNF: Tumor necrosis factor
TNFR: TNF-receptors
FasL: Fas ligand
CDKs: Cyclin-dependent kinases
TGFB: Transforming Growth Factor-Beta
FAS: Fibroblast associated antigen
GADD: Growth arrest and DNA damage-inducible
DAPK: Death-associated kinase
DRs: Death receptors
FADD: Fas-associated death domain
EGF: Epidermal growth factor
EGFR: EGF receptor
IRE1α: Inositol-requiring transmembrane kinase/endoribonuclease 1α
YAP: Yes-associated protein
Notch1-IC: Notch1 intracellular domain
HCC: Hepatocellular carcinoma
CRC: Colorectal cancer
GC: Gastric cancer
DDP: Cisplatin
XIAP: X chromosome-linked inhibitor of apoptosis
HB: Hepatoblastoma
HD-SB: Hedyotis diffusa-Sculellaria barbata
Tan I: Tanshinone I
TNM: Tumor-node-metastasis
GSK-3β: Glycogen synthase kinase 3β
EMT: Epithelial-mesenchymal
CC: Cervical cancer
OC: Ovarian cancer
BCa: Bladder cancer
PC: Prostate cancer
EC: Endometrial cancer
OSCC: Oral squamous cell carcinoma
HNSCC: Head and neck squamous cell carcinoma
ESCC: Esophageal squamous cell carcinomas
AML: Acute myeloid leukemia
CLL: Chronic lymphocytic leukemia
DLBCL: Diffuse large B-cell lymphoma
SCLC: Small-cell lung cancer
NSCLC: Non-small-cell lung cancer
LUAD: Lung adenocarcinoma
BC: Breast cancer
OS: Osteosarcoma
PTC: Papillary thyroid cancer
NFPAs: Nonfunctioning pituitary adenomas
LC: Laryngeal carcinoma

## 1 Introduction

### 1.1 Circular RNAs and cancer

Globally, cancer is a leading cause of death. According to predictions, the number of deaths from various types of cancer will increase due to population growth and adaptation to changing lifestyle behaviors [1,2]. Gene alterations, especially those in proto-oncogenes and tumor suppressors, result in cellular proliferation, tissue destruction, invasion, and metastasis [3]. Some processes make cells unresponsive to apoptotic signals, leading to the transformation of a normal cell into a cancer cell [4].

Less than 2 % of human genes encode proteins, indicating that noncoding RNAs constitute most of the transcriptome [5]. Noncoding RNAs (ncRNAs), including micro RNAs (miRNAs) and circular RNAs (circRNAs), were formerly considered junk materials. Nonetheless, their involvement in cell biology is now recognized to be both physiological and pathological [5]. Since there is no polyadenylated tail or 5′ to 3′ polarity to be attacked, circular RNAs have a ring-shaped structure and are resistant to exonucleases. Their 3′ and 5′ sections are joined by a covalency bond [6,7]. Consequently, circRNA stability may make it a more effective indicator than other noncoding RNAs [8,9].

**Fig. 1:**
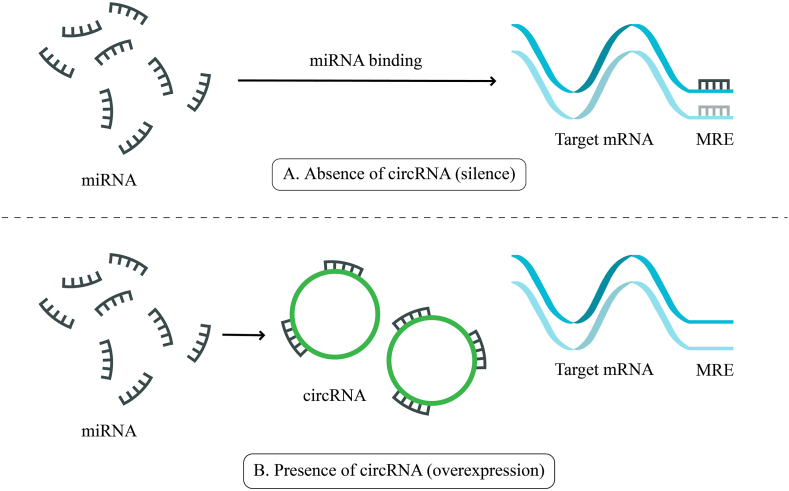
**The function of competitive endogenous RNA network.** (A) In the absence of circRNA, miRNA binds to the MRE (miRNA response elements) region of mRNA and prevents mRNA expression. (B) In the presence of circRNA, circRNA sponges miRNA and inhibits its function to induce mRNA expression level.

CircRNAs perform various biological tasks, but their primary role is to sponge microRNAs and stifle miRNA activity. Mechanistically, miRNAs attach to sequences called miRNA binding sites on circRNAs [[10], [11], [12]]. In the 3′-UTR region of downstream mRNAs, miRNAs bind to miRNA response elements (MREs) and prevent translation [13]. The interaction between circRNA and its downstream target miRNAs and mRNAs is referred to as a competitive endogenous RNA (ceRNA) network [13], as demonstrated in Fig. 1. In summary, complimentary sequences on circRNAs bind to miRNAs, indicating a potential function in controlling mRNA expression [13,14]. Furthermore, they can also take part in various physiological and pathological processes through diverse mechanisms. For instance, it was discovered that circRNAs control gene expression by influencing alternative splicing through RNA-mediated interaction. These unique RNAs also have the potential to interact with RNA-binding proteins (RBPs) as protein scaffolds or antagonists. Despite the fact that circRNAs have been categorized as "non-coding" elements, the majority of circRNAs are distributed primarily in the cytoplasm, which raises the possibility that they may translate once their internal ribosome entry site (IRES) is triggered [15,16].

Additionally, circRNAs may be involved in the development of tumors [12,17]. Dysregulation of circRNAs is correlated with cancer development in various types of cancer [18], such as colorectal cancer [19,20], ovarian cancer [21,22], and gastric cancer [23,24]. CircRNA expression profiles vary depending on the type of tumor and are tissue-specific, changing during different phases of cell development [5,7]. It has been discovered that the concentration of circRNAs can vary, which may impact the apoptosis rate [13]. Additional research has shown that modifications in circRNA levels also affect the regulation of cell signaling pathways [25,26]. In this review, we present a summary of studies on the role of ceRNA networks in cellular signaling pathways that regulate apoptosis.

### 1.2 Apoptosis

Apoptosis, a form of programmed cell death, is a natural biological mechanism that tissues use to maintain tissue homeostasis, control cell numbers, and inhibit tumor growth without causing inflammation [27,28]. Nucleus condensation, DNA fragmentation, cell shrinkage, dynamic membrane blebbing, and loss of adherence to the external matrix are characteristics of apoptosis [29]. In addition to pro and antiapoptotic factors, endogenous and exogenous factors play a role in this process [27].

Generally, two pathways can initiate apoptosis in a strictly regulated process: intrinsic and extrinsic. The intrinsic pathway can be activated by internal stimuli such as cell stress, DNA damage, developmental cues, withdrawal of survival factors, or hypoxia and can be regulated by Bcl-2 family proteins [29]. The extrinsic pathway is triggered by detecting extracellular stimuli, such as binding ligands to death receptors on the cell surface [30,31]. Both pathways activate caspases, as cysteine proteases involved in the process of apoptosis. They become activated by proteolytic cleavage and cleave numerous cellular substrates, to induce cell death [32]. Cytochrome *c* interacts with ATP and apoptotic protease activating factor-1 (Apaf-1) and activates procaspase-9 to trigger the caspase cascade and induce apoptosis [28].

In further review, the regulation of apoptosis involves multiple signaling pathways and molecules. These pathways work together to ensure that apoptosis occurs only when necessary to maintain tissue homeostasis and eliminate unwanted or damaged cells. The following are some of the fundamental molecular mechanisms and signaling pathways that regulate apoptosis.

### 1.3 Signaling pathways crosstalk with apoptosis

Death receptors (DRs) are transmembrane proteins that are members of the TNF (tumor necrosis factor) receptor superfamily. They can be induced by ligands such as FasL and TNF-α, which initiate the extrinsic apoptotic pathway [33].

The intrinsic pathway of apoptosis is modulated by proteins of the Bcl-2 family, which are divided into proapoptotic and antiapoptotic members. They cooperate to control cytochrome *c* release and the permeability of the outer membrane of mitochondria [4,34].

Research using tumor xenograft models and cell culture has shown that blocking STAT activation enhances apoptosis and reduces cell growth. Cell cycle progression is promoted by STAT3 through the activation of cyclin-dependent kinases (CDKs). It suppresses CDK inhibitors’ expression levels like p21 and overexpresses positive regulators, such as cyclin D. STAT5 protects cells against apoptosis by increasing Bcl-x transcription, which produces the antiapoptotic protein Bcl-xL [35,36]. The antiapoptotic feature of STAT3 is also related to its ability to induce Bcl-xL and Mcl-1 expression [37].

The TGF-β (Transforming Growth Factor-Beta) signaling pathway functions as a tumor suppressor in the early stages of cancer by inhibiting cell development, hence inducing apoptosis. TGF-β suppresses cell division by activating p21 and p15 and inhibiting c-Myc as the suppressor of these molecules [38].

TGF-β also causes apoptosis by regulating the expression of Bcl-2 family members, death receptor fibroblast-associated antigen (FAS), growth arrest and DNA damage-inducible (GADD) 45-β, death-associated kinase (DAPK), and caspases, resulting in both intrinsic and extrinsic apoptosis [39].

TNF-α is a proinflammatory cytokine that may paradoxically induce apoptosis or cell survival. TNF-α binds to TNF-receptors (TNFRs) 1 and 2. The TNFR1 receptor mainly transmits apoptotic signals [30]. TNF is probably the most significant apoptosis inducer [33].

Death receptors (DRs) are components of the tumor necrosis factor receptor superfamily. In the extrinsic pathway, another crucial adaptor protein is the Fas-associated death domain (FADD). The death-inducing signaling complex (DISC) activates procaspase-8, which activates procaspase-3, resulting in cell death. Caspase-8 induces Bid to activate the intrinsic pathway [40]. It is revealed that FADD can also regulate the NF-κB pathway [41].

PTEN may directly inhibit PI3K signaling and prevent Akt from binding to PI3K, which may play a role in the induction of apoptosis. PI3K/AKT pathway regulates multiple cellular processes through AKT, which is the central node of the pathway. PI3K phosphorylates and activates Akt by producing PIP3 [42]. The hyperactivation of AKT can lead to the induction of cell proliferation, resistance to apoptosis, tumor development, and resistance to cancer therapies. The tumor suppressor PTEN is downregulated, while the antiapoptotic component Bcl-2 is upregulated in response to activated PI3K/AKT signaling [43,44]. mTOR activity is regulated by growth factors that activate Akt [45].

Proliferation and differentiation are largely regulated by epidermal growth factor receptor signaling. EGF ligands that bind to EGFR1 and EGFR2 can cause apoptosis [46]. When EGFR binds to EGF, it enhances PTEN expression by activating Ref-1. Ref-1 can influence several biological processes, such as cell cycle arrest, apoptosis, and cell survival, because of its distinct structure [47].

The intricate pathway known as RAS (Rat Sarcoma) signaling can affect apoptosis regulation positively or negatively, depending on the type of cell and other factors [48]. By phosphorylating several apoptosis-regulating factors, including Bad, Bim, Mcl-1, caspase-9, and the contentious Bcl-2, the Ras/Raf/MEK/ERK cascade plays a significant role in the process of apoptosis [48].

Under some circumstances, the ERK1/2 kinases in the RAF/MEK/ERK signaling pathway can also serve as proapoptotic agents, and elevated ERK1/2 signaling can result in the death of cancer cells [49]. Renal cell death caused by cisplatin is mediated by ERK1/2 kinases, which function upstream of caspase-3 [50]. Multiple DNA-damaging agents induce apoptosis by upregulating p53 and modifying Bcl-2 family proteins via the MEK signaling pathway [51,52]. In cells that undergo cisplatin-induced apoptosis, ERK facilitates the activation of the p53 signaling pathway, which consequently induces the expression of Bax [53]. In renal tissues, the suppression of ERK attenuates TNF-α expression, caspase-3 activation, and apoptosis [54]. Additionally, ERK can enhance apoptosis by inhibiting survival signaling pathways, such as PI3K/AKT signaling [55].

Phosphorylated JNK translocates to the nucleus via the MEKK/SLK/JNK signaling pathway, where it phosphorylates and transactivates c-Jun, causing the formation of AP-1 [8,41]. Proapoptotic proteins, including TNF-α, FasL, and Bak, are among the various proteins whose transcription is regulated by AP-1 [56,57]. Ras and other oncogenes potently stimulate JNK activation [58].

A further function of JNK is to phosphorylate Bim and Bmf from their scaffold proteins to hinder Bcl-2 from having an antiapoptotic effect. In addition, JNK directly phosphorylates Bad and Bax to facilitate their translocation from their 14-3-3-mediated sequestering complex [59]. By phosphorylating Bad and Bim, JNK can directly target mitochondria; therefore, these proapoptotic proteins inhibit the function of antiapoptotic proteins like Bcl-2 and Bcl-xL [60].

It has been demonstrated that JNK and ERK activate p38 MAPK. When p38 activity is elevated, p38 appears to have proapoptotic properties [57].

Moreover, JNK, which is activated by death receptors, stimulates the extrinsic pathway by directly phosphorylating Bid. Bid is cleaved through phosphorylation, which results in the production of jBid and its translocation to mitochondria. Smac and Omi are released from mitochondria by both jBid and full-length Bid that JNK has phosphorylated. Suppression of XIAP (X-linked inhibitor of apoptosis) by Smac and Omi and downregulation of cIAP1 trigger the activation of executioner caspases 3 and 7, ultimately resulting in apoptosis [61]. XIAP inhibits apoptosis by blocking the activation and maturation of caspase-3, caspase-7, and caspase-9, all essential for initiating and triggering apoptosis [62].

According to Manel B. Hammouda's research, JNK is necessary for the expression of the JunD transcription factor, which cooperates with NF-κB to stimulate the expression of prosurvival genes, including cIAP-2, in fibroblasts [60].

Research from other sources indicates that JNK enhances cell survival by working in concert with NF-κB and JAK/STAT signaling. Additionally, JNK blocks TRAF2/IAP1 signaling by triggering the release of Smac/Diablo from mitochondria and activating caspase 8 as a result. ASK1 and JNK are activated by Inositol-requiring transmembrane kinase/endoribonuclease 1α (IRE1α) recruiting TRAF2, which in turn impedes antiapoptotic proteins like Bcl-2, Bcl-xL, and Mcl-1 [63].

JNK induces cell death in response to DNA damage through p53 phosphorylation, p73 stabilization, and p53/p73-dependent expression of the proapoptotic proteins Bax and Puma [60].

The proto‐oncogene YES1 is a member of SRC family kinases that plays a role in cell proliferation and suppresses apoptosis by triggering β-catenin signaling to elevate the level of cyclin D [64].

In mammals, a key element of the Hippo signaling system is Yes-associated protein (YAP) [65]. It has been shown that YAP's interaction with TEAD transcription factors may enhance the expression of genes that prevent apoptosis, including *COX-2* [66], *Survivin* [67], and *Glut1* [68]. According to reports, p73 is a transcriptional partner of YAP and increases the expression of a number of proapoptotic genes, including *p53AIP1* [69], *Bax* [70], *DR5* [71], and *PUMA* [72]. As a result, YAP can promote the expression of both pro- and antiapoptotic genes.

In a variety of cells, YAP exhibits antiapoptotic properties. For instance, in hepatocellular carcinoma cells, YAP decreases apoptosis by upregulating Jag-1 to activate Notch signaling [73]. Furthermore, Fbxw7-induced YAP ubiquitination and proteasomal degradation promote apoptosis in hepatocellular carcinoma cells. Conversely, YAP expression restoration partially reverses the effects of Fbxw7-induced cell death and growth arrest both *in vitro* and *in vivo* [74].

Akt and c-Abl phosphorylate YAP in response to stress brought on by significant DNA damage, which inhibits the induction of proapoptotic gene expression and eventually causes apoptosis [75]. YAP can cause apoptosis through binding p73 rather than TEAD, which causes the proapoptotic gene Bax to be upregulated [76].

β-catenin/T-cell factor-mediated transcription is activated by the Wnt/β-catenin signaling pathway, which prevents apoptosis [77]. Wnt-1 has the potential to suppress caspase-9 activity. Further research has demonstrated that Wnt signaling, which activates NF-κB or decreases GSK3-β (glycogen synthase kinase 3β), can minimize apoptosis and improve survival [78]. When β-catenin/Tcf transcription was inhibited, Wnt-1-mediated cell survival was prevented, and cells were susceptible to apoptotic triggers [28]. Truncated APC protein accumulation in mitochondria can cause attachment of these molecules to Bcl-2 proteins and raise their concentration in this organelle [78].

In human tumor cells, the Hedgehog (Hh) signaling pathway can prevent cell death by upregulating the antiapoptotic gene Bcl-2 [79]. The Hh pathway can enhance the rate of apoptosis through its transcriptional target gene rdx, which encodes an E3 ubiquitin ligase [80].

NF-κB signaling pathway is commonly thought to be antiapoptotic, however there are situations in which it can actually encourage apoptosis. In response to cell stress, NF-κB triggers apoptosis by accumulating RelA in nucleolus. RelA can promote apoptosis, and NF-κB activation is necessary for p53-mediated cell death [81]. Moreover, proapoptotic activity can be exhibited by IκBα, the subunit that prevents the activation of NF-κB. Ultimately, cytochrome *c* release resulting from NF-κB activation in the mitochondria can cause caspase cascades and programmed cell death [82].

**Fig. 2:**
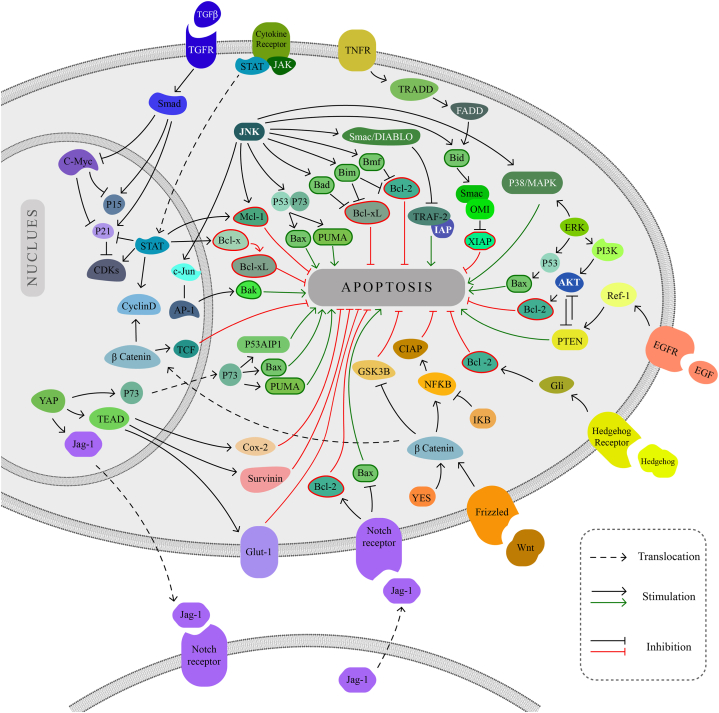
**Apoptosis regulation through cell signaling pathway molecules.** Various mechanisms modulate apoptosis. The pro-apoptotic members are shown in green, and the anti-apoptotic members are shown in red. Jag-1 interacts with the Notch receptor on the adjacent cell membrane to trigger Notch signaling pathway.

The notch signaling pathway interacts with other signaling pathways, such as p53, NF-κB, and PI3K-Akt pathways, and can control apoptosis through a variety of complex networks that include cell cycle, growth, and survival pathways [83]. Notch-1 blockade seems to control the expression of Bcl-2 family members by downregulating the antiapoptotic protein Bcl-2 and upregulating the proapoptotic protein Bax [84]. By inhibiting the apoptosis signal-regulating kinase 1 enzyme, the Notch1 intracellular domain (Notch1-IC) can stop oxidative stress-induced cell death [85]. In neural progenitor cells, Notch activation induces apoptotic cell death through a p53-dependent mechanism [86]. The complex interplay between these signaling pathways and their regulatory mechanisms on apoptosis is shown in Fig. 2.

In conclusion, circRNAs sponge certain miRNAs to regulate the expression of their target mRNAs. These mRNAs directly or indirectly affect cellular signaling pathways or specific apoptosis-related molecules to modulate cell apoptosis. Studies have demonstrated that dysregulated expression of circRNAs, either as oncogenes or tumor suppressors, could contribute to the pathogenesis of cancers and apoptosis resistance through disruption of the balance of pro and antiapoptotic molecules, in addition to alterations in the expression levels of miRNAs and targeted mRNAs.

Researchers have mostly mentioned these circRNAs as insights for the probable clinical treatment of particular cancers, although it faces some challenges. For instance, the full spectrum of their functions in different cellular contexts remains unclear and regulatory factors involved in these processes are not fully clarified. In this paper, we propose to review the role of ceRNA networks in cellular signaling pathways modulating apoptosis in human cancers for a more comprehensive look at probable treatment methods. In other words, one approach for cancer treatment is to drive cells toward apoptosis. In the following section, we introduce different ceRNA networks that ultimately play critical roles in cell death via various signaling pathways.

## 2 Overview of ceRNA research in cancers

### 2.1 Gastrointestinal cancers

#### 2.1.1 Liver cancer

There are different subtypes of liver carcinoma, and hepatocellular carcinoma (HCC) is the most common [87].

The significant upregulation of the expression of circ_0067934 and circ_104348, which act as oncogenes that sponge miR-1324 and miR-187-3p, respectively, has been observed in HCC cells and tissues. Both of these circRNAs can promote the Wnt/β-catenin pathway to induce the progression of cancer [88,89].

A target gene of miR-1324 is FZD5, and downregulating circ_0067934 suppresses the protein levels of FZD5, nuclear β-catenin, and Cyclin D1. RTKN2 is the target of miR-187-3p [88,89].

On the other hand, the expression of circRNA-ITCH was reduced in HCC cell lines, and its overexpression might act as a tumor suppressor by sponging miR-7 or miR-214 to regulate the level of ITCH, which could inhibit the expression of c-Myc and cyclin D1 and suppress the Wnt/β-catenin signaling pathway [90].

Upregulated expression of circ9119 and circ_0072088 acted as endogenous sponges of miR-26a and miR-375, respectively, in HCC specimens and cell lines. JAK1 is targeted by miR-26a, and JAK2 is the target of miR-375; both of these factors consequently phosphorylate STAT3, leading to its nuclear translocation and contributing to the activation of the JAK/STAT pathway and decreasing apoptosis [91,92]. Downregulating circ9119 contributed to a reduction in the expression level of Bcl-2, while Bax expression remained unchanged [91].

Low expression of circCCNB1 resulted in upregulation of miR-106b-5p to inhibit GPM6A expression, which modulated the expression level of DYNC1I1 in HCC cells and cancer tissues. DYNC1I1 inhibited apoptosis, participated in the regulation of the cell cycle, and acted as an activator of the AKT/ERK signaling pathway [87].

CircKIF5B plays an oncogenic role in HCC, and its silencing reduces cancer progression by sponging miR-192 and miR-215, thus decreasing the expression of XIAP, which is a protein that prevents apoptotic cell death [93].

Circ_0036412 triggers proliferation and inhibits cell cycle arrest at the G2/M phase in HCC cells. Despite the findings of other studies on ceRNA networks, this study indicated that circ_0036412 could not affect HCC cell apoptosis. Circ_0036412 upregulates GLI2 expression by sponging miR-579-3p and has been shown to diminish the activity of the Hedgehog pathway [94].

80 % of pediatric liver cancers are related to hepatoblastoma (HB). HB patients with high circHMGCS1 expression had shorter overall survival. CircHMGCS1 sponges miR-503-5p to modulate IGF2 and IGF1R expression and affects the PI3K-Akt signaling pathway [95].

In another study, circSETD3 was revealed to be underexpressed in HB tissues and cell lines. Upregulation of circSETD3 increased the apoptosis rate in HB cells. circSETD3 sponged miR-423-3p to regulate Bim. The level of the proapoptotic protein Bcl-2 was also increased, but the level of the antiapoptotic protein Bax was reduced by circSETD3 upregulation [96].

#### 2.1.2 Colorectal cancer (CRC)

Globally, colorectal cancer (CRC) is one of the most common malignancies with a high mortality rate [97].

Several circRNAs have been shown to act as oncogenes in the progression of CRC. These genes include circRASSF2 [98], circ-IGF1R [99], circIFT80 [100,101], circRNA_100290 [102], circCCT3 [20], circ_0006174 [19], and circAGFG1 [97]; The ceRNA networks are fully stated in Table 1. Upregulation of these circRNAs ultimately contributes to increasing the expression of downstream genes by sponging their target miRNAs to activate the Wnt/β-catenin pathway.

**Table 1:** Gastrointestinal cancers.

CircRNA	Expression	miRNA	Target mRNA	Signaling pathway	Ref
**Liver cancer**					
circ_0067934	up-regulated	miR-1324	FZD5	Wnt/β-catenin	[88]
hsa_circRNA_104348	up-regulated	miR-187–3p	RTKN2	Wnt/β-catenin	[89]
circRNA-ITCH	down-regulated	miR-7 miR-214	ITCH	Wnt/β-catenin	[90]
circ9119	up-regulated	miR-26a	JAK1	JAK1/STAT3	[91]
circ_0072088	up-regulated	miR-375	JAK2	JAK2/STAT3	[92]
circCCNB1	down-regulated	miR-106b-5p	GPM6A	Akt/ERK	[87]
circKIF5B	up-regulated	miR-192 miR-215	XIAP	–	[93]
circ_0036412	up-regulated	miR-579-3p	GLI2	Hedgehog	[94]
circHMGCS1	up-regulated	miR-503-5p	IGF2, IGF1R	PI3K-Akt	[95]
circSETD3	down-regulated	miR-423-3p	Bim	–	[96]
**Colorectal cancer**					
hsa_circ_0000523 (circ_006229)	down-regulated	miR-31	DDK1	Wnt/β-catenin	[103]
circRASSF2	up-regulated	miR-195-5p	FZD4	Wnt/β-catenin	[98]
circ-IGF1R	up-regulated	miR-362-5p	HMGB3	Wnt/β-catenin	[99]
circIFT80	up-regulated	miR-142, miR-568, miR-634	β-catenin (CTNNB1)	Wnt/β-catenin	[100]
hsa_circ_0001666	down-regulated	miR-576-5p	PCDH10	Wnt/β-catenin	[104]
circRNA_100290	up-regulated	miR-516b	FZD4	Wnt/β-catenin	[102]
circCCT3	up-regulated	miR-613	WNT3, VEGFA	Wnt	[20]
circ_0006174	up-regulated	mir-1205	CCBE1	Wnt	[19]
circAGFG1	up-regulated	miR-4262, miR-185-5p	YY1	Wnt/β-catenin	[97]
hsa-circRNA-0067835 (circIFT80)	up-regulated	miR-370-3p	WNT7B	Wnt	[101]
hsa_circ_0039933	up-regulated	miR-204-5p	wnt11	Wnt	[105]
circRNA_0074027	up-regulated	miR-525-3p	–	p53/EMT	[106]
circ_0011385	up-regulated	miR-330-3p	MYO6	MEK1/2/ERK1/2	[107]
circ_0004585	up-regulated	miR-338-3p	ZFX	MEK/ERK	[108]
circ-LECRC	down-regulated	miR-135b-5p	KLF4	YAP1	[109]
circEPB41L2	down-regulated	miR-21-5p, miR-942-5p	–	PTEN/Akt	[110]
circPACRGL	up-regulated	miR-142-3p, miR-506-3p	TGF-β1	TGF-β1	[111]
circNSUN2	up-regulated	miR-296-5p	STAT3	STAT	[112]
**Colon cancer**					
circCDYL	down-regulated	miR-150-5p	–	PI3K/Akt, JAK2/STAT5	[114]
**Gastric cancer**					
circ-SFMBT2	up-regulated	miR-885-3p	CHD7	Wnt/β-catenin	[115]
circRNA_0044516	up-regulated	miR-149	–	Wnt1/β-catenin	[116]
circMAN2B2	up-regulated	miR-145	–	PI3K/Akt & JNK	[117]
circPVT1	up-regulated	miR-152-3p	HDGF	PI3K/Akt	[23]
circRNA_100395	down-regulated	miR-142-3p	–	PI3K/Akt	[119]
circ_0006089	up-regulated	miR-143-3p	PTBP3	PI3K/Akt	[118]
hsa_circ_0006282	down-regulated	miR-136-5p	PTEN	PTEN/Akt	[120]
circ_0067997	up-regulated	miR-515-5p	XIAP	XIAP	[121]
circRNA_100876	up-regulated	miR-665	YAP1	YAP1	[122]
hsa_circ_0017728	up-regulated	miR-149	IL-6	STAT3	[123]
hsa_circ_006100	up-regulated	miR‐195	GPRC5A	GPRC5A/EGFR	[24]
circCCDC66	up-regulated	miR-618	Bcl-2	–	[124]
**Pancreatic cancer**					
circ_0007534	up-regulated	miR‐625, miR‐892b	Bcl‐2/caspase‐3	–	[126]
circEIF6	up-regulated	miR-557	SLC7A11	PI3K/Akt	[127]

CRC progression and tumorigenesis are also associated with the downregulation of the tumor suppressors hsa_circ_0000523 (or circ_006229) and hsa_circ_0001666, by suppressing cell apoptosis via miR-31 and miR-576-5p absorption, respectively, and subsequent activation of the Wnt/β-catenin signaling pathway. Mir-576-5p overexpression inhibited PCDH10 gene, which suppresses the Wnt/β-catenin signaling pathway [103,104]. It is also revealed that HD-SB (Hedyotis diffusa-Sculellaria barbata) downregulates wnt11 expression through the hsa_circ_0039933/hsa-miR-204-5p axis, finally inhibiting the Wnt signaling pathway [105].

Overexpressing circRNA0074027 in CRC tissues might regulate the p53/EMT signaling pathway by sponging miR-525-3p. CircRNA_0074027 downregulation increased p53 and Bax and decreased Bcl-2 protein expression [106].

MEK1/2/ERK1/2 signaling pathways were also found to be regulated in CRC tissues or cells by overexpressing circ_0011385 and MYO6, while miR-330-3p was underexpressed [107].

In CRC, circ_0004585 sponges miR-338-3p to upregulate ZFX. Circ_0004585 activated MEK/ERK pathway by inducing ZFX [108].

CircLECRC, a Yes1 associated transcriptional regulator (YAP1)-derived circRNA, expression was markedly reduced in CRC. This circRNA could function as a sponge for miR-135b-5p to upregulate KLF4 and induce CRC cell apoptosis in a KLF4-dependent manner. Overexpression of YAP1 decreased the expression of downstream genes, such as EGFR, MYC, BIRC5, and CTGF. Thus, circ-LECRC restrains the progression of CRC to hamper the hyperactivation of YAP1 signaling [109].

The expression of exosomal circEPB41L2 in CRC cells was reduced, while modulated the PTEN/AKT signaling pathway by sponging miR-21-5p and miR-942-5p [110].

CircPACRGL increased the expression of TGF-β1 by sponging miR-142-3p/miR-506-3p in CRC cells [111].

Wei Han indicated that circNSUN2 silencing in CRC cells hinders progression and induces apoptosis. CircNSUN2 sponges miR-296-5p, which regulates STAT3 [112].

#### 2.1.3 Colon cancer

The incidence of colon cancer has increased in young people in recent years [113]. In colon cancer tissues, the expression level of circCDYL was decreased, while miR-150-5p was overexpressed. circCDYL induced apoptosis in colon cell lines by downregulating c-Myc and cyclin D1, inducing p53, and cleaving PARP and caspase-3. CircCDYL functions as a tumor suppressor in cell development and reduces the PTEN level and phosphorylation of PI3K, AKT, JAK2, and STAT5 [114].

#### 2.1.4 Gastric cancer (GC)

It is still a significant health problem, particularly for advanced gastric cancer (GC), which has a high mortality rate and poor response to treatment after curative resection [24].

The Wnt/β-catenin signaling pathway in GC was found to be activated by upregulating circ-SFMBT2 sponging miR-885-3p [115] and circRNA_0044516 sponging miR-149 [116]. Circ-SFMBT2 regulates CHD7 expression, and its knockdown leads to a reduction in Wnt1 and Catenin levels, while inducing apoptosis [115,116].

High levels of circMAN2B2 and circ_0006089, and low levels of circRNA_100395 and hsa_circ_0006282 were found in GC tissues compared to paracancerous tissues. Inducing the reverse expression of these circRNAs significantly reduced the viability of GC cell lines, whereas increased their apoptosis and decreased the levels of phosphorylated AKT and PI3K [[117], [118], [119], [120]].

CircMAN2B2 silencing contributed to the deactivation of PI3K and AKT pathways, activation of JNK, induction of caspase cleavage (−3 and −9), downregulation of MMPs (−2 and −9), and upregulation of miR-145 [117]. Circ_0006089 sponges miR-143-3p to control the expression of PTBP3 [118].

Besides, in DDP-resistant GC cells, the knockdown of circPVT1 reduced the expression level of HDGF by sponging miR-152-3p, and hampered the PI3K/AKT signaling pathway and induced apoptosis [23].

CircRNA_100395 and hsa_circ_0006282, as tumor suppressors in GC that sponge miR-142-3p and hsa-miR-136-5p, respectively, contribute to the induction of PTEN protein expression to hinder the PI3K/AKT pathway [119,120].

The activation of XIAP was induced by the overexpression of circ_0067997, which sponges miR-515-5p in GC and was related to a low rate of GC patients' overall survival [121]. Upregulation of circRNA_100876 might modulate YAP1 expression levels by sponging miR-665 [122]. Hsa_circ_0017728 sequesters miR-149 to regulate the IL-6/STAT3 signaling pathway in GC cells [123].

Another probable oncogene in GC cells is hsa_circ_006100, which induces cancer progression by inhibiting miR‐195. GPRC5A is a target of miR‐195. Min Liang showed that hsa_circ_006100 knockdown decreased PCNA expression, while increased miR-195 and Bcl-2 expression as a result of GPRC5A/EGFR signaling [24].

In GC patients receiving CDDP-based chemotherapy, circCCDC66 was upregulated, and Bcl-2 expression was increased by sponging miR-618 to reduce the apoptotic rate [124].

#### 2.1.5 Pancreatic cancer

The most frequent malignant tumor of the pancreas is called pancreatic ductal adenocarcinoma (PDAC) [125]. In PDAC cells, circ_0007534 upregulation sponges miR‐625 and miR‐892b to hamper cell apoptosis through the Bcl‐2/caspase‐3 pathway [126].

PI3K/AKT pathway was shown to be activated by circEIF6 overexpression in pancreatic tumor tissues and cell lines. CircEIF6 regulates SLC7A11 via miR-557 sponging [127].

A comprehensive summary of the ceRNA networks related to gastrointestinal cancers is shown in Table 1.

### 2.2 Genitourinary cancers

#### 2.2.1 Cervical cancer (CC)

Cervical cancer (CC) is a frequent gynecological disease primarily caused by an infection with the human papillomavirus [128].

In CC tissues, the expression levels of circAMOTL1 and hsa_circ_CSPP1, which regulate SIK2 and ITGB1, respectively, were increased, while apoptosis was decreased [128,129].

CircAMOTL1 overexpression led to a decrease in the expression of miR-526b and the induction of AKT signaling pathway by regulating SIK2 [128]. PI3K-Akt signaling pathway is also activated by the upregulation of hsa_circ_CSPP1, which sponges miR-361-5p in CC [129].

Upregulation of circRNA MYLK which sponges miR-1301-3p led to RHEB expression induction, mTOR signaling activation, and apoptosis inhibition in CC cells [130].

The sponge of miR-145-5p by circ_0000392, increases the radiosensitivity of CC cells and might be due to the CRKL/ERK signaling pathway [131].

#### 2.2.2 Ovarian cancer (OC)

Women frequently develop ovarian cancer (OC), an extremely life-threatening disease. Since ovarian disorders grow deep within the pelvis, they typically have atypical symptoms and are usually diagnosed when they advance to distant metastases [22].

Circ-ABCB10 indirectly induces the expression of Capn4 by sponging miR-1271 to regulate Wnt/β-catenin pathway in epithelial OC. MiR-1271 can decrease the expression of Capn4, FOXR2, regulator of G-protein signaling 17, and spindlin 1 proteins downstream [132].

A tumor suppressor in OC cells is hsa_circ_0001445 (hcR1445), which reduces SFRP1 expression by targeting miR-576-5p. Low expression of SFRP1 activated Wnt/β-catenin signal transduction in OC cells [22].

Circ9119, which sponges miR-21, was expressed at low levels in OC cells and targeted PTEN-Akt pathway. Jianming Gong also reported that circ9119 overexpression triggered apoptotic cascade cleavage, activity, and expression, while overexpressing miR-21 suppressed PTEN expression, which influenced the phosphorylation and nuclear localization of Akt [21].

On the other hand, circSETDB1 acted as an oncogene in serous ovarian cancer progression by sponging miR-129-3p to regulate MAP3K3 expression. In the MAPK pathway, MAP3K3 functions as an upstream regulator [133].

Circ-PGAM1 sequesters miR-542-3p to indirectly upregulate CDC5L, which is an oncogene in OC cells. CDC5L promotes PEAK1 transcription by binding to its promoter. Upregulation of PEAK1 could trigger ERK1/2 and JAK2 signaling pathways [134].

The upregulation of circ_0067934 in OC samples was related to the stage of tumor and lymph node metastasis. Circ_0067934 sponges miR-545-3p and increases the translation of PPA. PPA1 overexpression reduced the phosphorylation of JNK, a proapoptotic signaling pathway [135].

#### 2.2.3 Bladder cancer (BCa)

Approximately 70 % of patients with bladder cancer (BCa) are diagnosed with a noninvasive disease that is physically excisable, while the remaining patients are at risk of developing muscle-invasive bladder cancer and having it migrate to other organs [136].

Several signaling pathways are detected to be affected by different ceRNA networks that function in BCa.

Chen et al. showed that in BCa cell lines and tissues, circ_0000326 was overexpressed and could promote PI3K/AKT signaling pathway via miR-338-3p suppression and indirectly induce the expression of ETS1 [137]. CircSEMA5A diminishes miR-330-5p to induce ENO1 expression and initiates the Akt and β-catenin signaling pathways [138].

CircCEP128 overexpression by sponging miR-145-5p upregulates MYD88 and downstream proteins in the MAPK signaling pathway [136]. CircINTS4 triggers NF-kB signaling, inhibiting the P38 MAPK signaling pathway through CARMA3 in BCa cells. Mechanistically, circINTS4 sponges miR-146b to rescue the expression of CARMA3 [139]. CircRNA-MYLK sequesters miR-29a to upregulate VEGFA, facilitating VEGF/VEGFR2 and the subsequent Ras/ERK signaling pathway in BCa progression [140]. Circ_001418 overexpression upregulated the expression of EphA2 and cytochrome C proteins and diminished FADD protein expression by sponging miR-1297 [141].

#### 2.2.4 Prostate cancer (PC)

Prostate cancer (PC) is a severe disease that impacts men's health all over the world [142].

In PC, circ_0057553 inhibited miR-515-5p to upregulate YES1. YES proto-oncogene 1 (YES1) belongs to the nonreceptor protein tyrosine kinase family and is involved in numerous processes that lead to tumor formation [143].

#### 2.2.5 Endometrial cancer (EC)

Endometrial cancer (EC) is a clinically diverse disease, and it is becoming evident that this variability may be due to the complexity of the underlying molecular dysfunctions [144].

In EC tumor tissues and cells, circTNFRSF21 was overexpressed. CircTNFRSF21 promotes MAPK13/ATF2 signaling pathway by sponging miR-1227 [145].

Circ_0109046 overexpression sponges miR105 to induce high SOX9 expression, contributing to Wnt/β-catenin signaling activation and apoptosis inhibition in EC [146].

Table 2 refers to the networks of genitourinary cancers.

**Table 2:** Genitourinary cancers.

CircRNA	Expression	miRNA	Target mRNA	Signaling pathway	Ref
**Cervical cancer**					
circAMOTL1	up-regulated	miR-526b	SIK2	Akt	[128]
circMYLK	up-regulated	miR-1301-3p	RHEB	mTOR	[130]
hsa_circ_CSPP1	up-regulated	miR-361-5p	ITGB1	PI3K/Akt	[129]
circ_0000392	up-regulated	miR-145-5p	CRKL	MAPK	[131]
**Ovarian cancer**					
circ-ABCB10	up-regulated	miR-1271	Capn4	Wnt/β-catenin	[132]
hsa_circ_0001445 (hcR1445)	down-regulated	miR-576-5p	SFRP1	Wnt/β-catenin	[22]
circ9119	down-regulated	miR-21	PTEN	PTEN/Akt	[21]
circSETDB1	up-regulated	miR-129-3p	MAP3K3	MAP3K3	[133]
circ-PGAM1	up-regulated	miR-542-3p	CDC5L	ERK1/2 & JAK2	[134]
circ_0067934	up-regulated	miR-545-3p	PPA1	JNK	[135]
**Bladder cancer**					
circ_0000326	up-regulated	miR-338-3p	ETS1	PI3K/Akt	[137]
circCEP128	up-regulated	miR-145-5p	MYD88	MAPK	[136]
circSEMA5A	up-regulated	miR-330-5p	ENO1	Akt & β-catenin	[138]
circINTS4	up-regulated	miR-146b	CARMA3	NF- κB & P38 MAPK	[139]
circRNA-MYLK	up-regulated	miR-29a	VEGFA	Ras/ERK	[140]
circ 001418	up-regulated	miR-1297	EphA2	cytochrome *c* & FADD	[141]
**Prostate cancer**					
circ_0057553	up-regulated	miR-515-5p	YES1	–	[143]
**Endometrial carcinoma**					
circTNFRSF21	up-regulated	miR-1227	MAPK13	MAPK13/ATF2	[145]
circ_0109046	up-regulated	miR-105	SOX9	Wnt/β-catenin	[146]

### 2.3 Squamous cell carcinomas

#### 2.3.1 Oral squamous cell carcinoma (OSCC)

The most prevalent kind of oral cancer, oral squamous cell carcinoma (OSCC), has an unfavorable prognosis and a high mortality rate.

Circ_0005050 was upregulated in OSCC cells. This circRNA targets miR-23a-3p and miR-625-5p to indirectly increase STAT3, leading to the activation of the JAK/STAT3 signaling pathway [147].

#### 2.3.2 Head and neck squamous cell carcinoma (HNSCC)

The most of head and neck malignancies arise from the mucosal epithelium of the oral cavity, pharynx, and larynx and are referred to as head and neck squamous cell carcinoma (HNSCC) [148].

In HNSCC cells, circ_0000052 is upregulated and sponges miR-382-3p to raise PD-L1 expression. PD-L1 reduction had an association with the activation of caspase-3/7. Increased PD-L1 levels in HNSCC could result from the IFN-γ/JAK2/STAT1 signaling pathway [149].

#### 2.3.3 Esophageal squamous cell carcinoma (ESCC)

Almost 90 % of esophageal cancers are esophageal squamous cell carcinomas (ESCCs), which have an inferior prognosis and a high fatality rate [37].

**Fig. 3:**
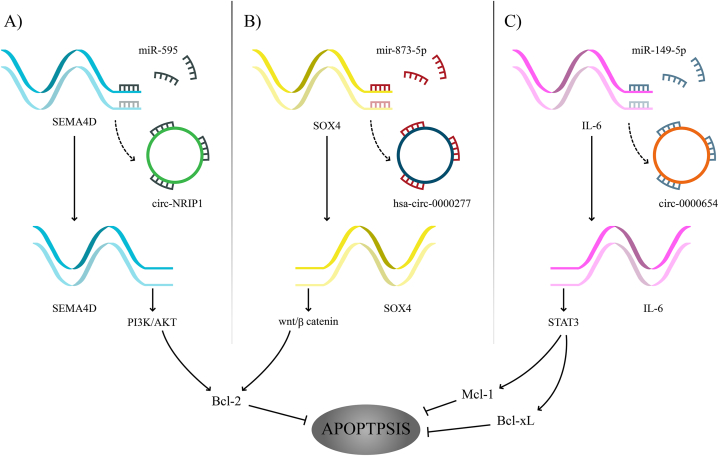
**The apoptotic mechanisms of cell signaling-associated ceRNA networks in esophageal squamous cell carcinoma.** (A) Circ_NRIP1 sponges miR-595 to indirectly trigger the PI3K/AKT pathway by upregulating SEMA4D. (B) Hsa_circ_000277 promotes SOX4 expression by sponging miR-873-5p to activate Wnt/β-catenin pathway. (C) Circ_0000654 acts as a sponge of miR-149-5p to overexpress IL-6 and induce STAT3 expression. Cell signaling pathways may influence the anti-apoptotic molecules to facilitate cell cycle progression and inhibit apoptosis.

Fig. 3 illustrates the apoptotic mechanisms of cell signaling-associated ceRNA networks in esophageal squamous cell carcinoma.

In ESCC, circ_NRIP1 overexpression upregulated SEMA4D by sponging miR-595. The oncogenic role of circ_NRIP1 is to trigger the PI3K/AKT signaling pathway. Low expression of circ_NRIP1 in ESCC cells contributed to an increase in the apoptosis rate and the expression of E-cadherin, while the expression of Snail was decreased [150].

Hsa_circ_0000277 induced SOX4 expression through sponging miR-873-5p to trigger Wnt/β-catenin signaling pathway [151].

Circ_0000654 was markedly increased in ESCC tissues and cell lines, and its high expression was strongly related to an elevated T stage and local lymph node metastasis in patients with ESCC. Circ_0000654 sequesters miR-149-5p to indirectly promote the IL-6/STAT3 signaling pathway [152].

The squamous cell carcinoma-related networks are summarized in Table 3.

**Table 3:** Squamous cell carcinoma.

CircRNA	Expression	miRNA	Target mRNA	Signaling pathway	Ref
**Oral squamous cell carcinoma (OSCC)**					
circ_0005050	up-regulated	miR-23a-3p, miR-625-5p	STAT3	JAK/STAT3	[147]
**Head and neck squamous cell carcinoma (HNSCC)**					
circ_0000052	up-regulated	miR-382-3p	PD-L1	IFN-γ/JAK2/STAT1	[149]
**Esophageal squamous cell carcinoma (ESCC)**					
circ_NRIP1	up-regulated	miR-595	SEMA4D	PI3K/Akt	[150]
hsa_circ_0000277	up-regulated	miR-873-5p	SOX4	Wnt/β-catenin	[151]
circ_0000654	up-regulated	miR-149-5p	IL-6	STAT3	[152]

### 2.4 Blood system cancers

#### 2.4.1 Acute myeloid leukemia (AML)

Acute myeloid leukemia (AML) is a bone marrow disease resulted from genetic alterations in blood cell precursors that cause an excess of clonal myeloid stem cells [153].

The JAK/STAT signaling pathway in AML cells is found to be activated as a result of circ_0104700 overexpression, which sponges miR-665 to upregulate MCM2 expression [154].

#### 2.4.2 Chronic lymphocytic leukemia (CLL)

Chronic lymphocytic leukemia (CLL) is defined by a marked increase in dysfunctional CD5^+^CD19^+^CD23^+^ B cells, which is caused by irregular cell proliferation and apoptosis. In CLL cells, upregulation of circ-CBFB led to an increase in FZD3 expression by sponging miR-607, finally resulting ultimately in Wnt/β-catenin pathway activation [155].

#### 2.4.3 Diffuse large B-cell lymphoma

Diffuse large B-cell lymphoma (DLBCL) is the most prevalent type of aggressive non-Hodgkin lymphoma from the germinal center [156]. In DLBCL tissues and cell lines, circ_0000877 was overexpressed and could indirectly upregulate MAP4K4 by targeting miR-370-3p. Reduction of MAP4K4 hampered Hippo pathway initiation [157].

Table 4 includes axes of blood system cancers.

**Table 4:** Blood system cancers.

CircRNA	Expression	miRNA	Target mRNA	Signaling pathway	Ref
**Acute myeloid leukemia**					
circ_0104700	up-regulated	miR-665	MCM2	JAK/STAT	[154]
**Chronic lymphocytic leukemia**					
circ-CBFB	up-regulated	miR-607	FZD3	Wnt/β-catenin	[155]
**Diffuse large B-cell lymphoma**					
circ_0000877	up-regulated	miR-370-3p	MAP4K4	Hippo	[157]

### 2.5 Other cancers

#### 2.5.1 Lung cancer

Lung cancer is one of the most common types of cancer and causes the majority of cancer-related deaths [158]. It can be divided into two primary subtypes: small cell lung cancer (SCLC) and non-small cell lung cancer (NSCLC). This classification is based on the appearance of cells under a microscope [159,160]. One particular subtype of non-small cell lung cancer is lung adenocarcinoma (LUAD) [161].

**Fig. 4:**
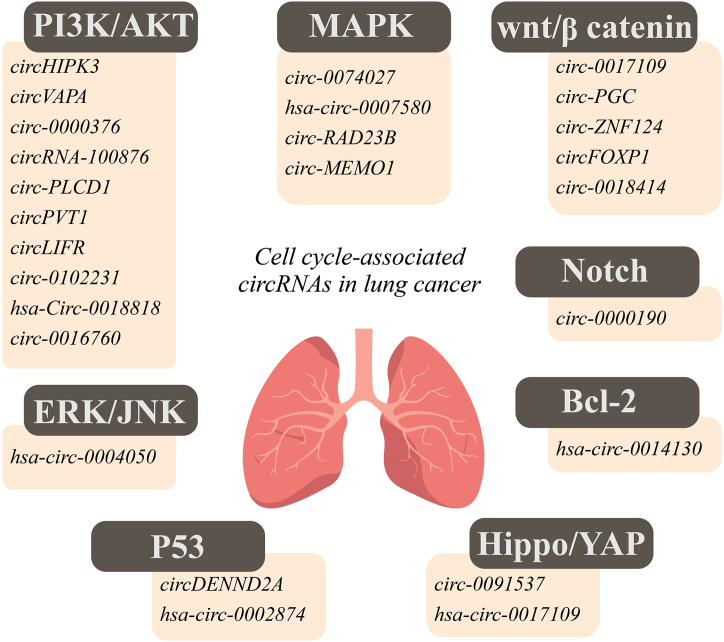
Cell cycle-associated circRNAs in lung cancer.

CircRNAs associated with the cell cycle in lung cancer are mentioned in Fig. 4. In lung cancer, circHIPK3 was revealed to promote cancer progression by sponging miR-381-3p and modulating the AKT/mTOR signaling pathway. CircHIPK3 knockdown suppressed cell proliferation, migration, and glycolysis, while increasing the apoptosis rate of lung cancer cells [25]. Luteolin inhibited Notch-1 signaling pathway in lung cancer by regulating circ_0000190 to sponge miR-130a-3p [162].

In small-cell lung cancer (SCLC) cell lines and clinical tissues, circVAPA was overexpressed to raise the IGF1R expression via sequestering miR-377-3p and miR-494-3p. Elevated levels of IGF1R lead to PI3K/AKT signaling pathway activation to enhance SCLC development. Following circVAPA knockdown in SCLC cells, the levels of the proteins p21 and cleaved PARP were significantly increased [163].

The ceRNA network could affect several cell signaling pathways to modulate apoptosis in non-small cell lung cancer (NSCLC).

It has been investigated that in NSCLC cells overexpressing circ_0017109, circ-PGC and circ-ZNF124 by sponging miR-671-5p, miR-532-3p, miR-498 could positively regulate FZD4, FOXR2 and YES1 expression, respectively to activate Wnt/β-catenin signaling pathway [26,158,164].

Circ_0017109 downregulation induced the cleavage of caspase-3, whereas reduced Bcl-2. FZD could modulate the Wnt/β-catenin signaling as an upstream regulator, therefor its downregulation, as a result of circ_0017109 suppressing, decreased the level of β-catenin, non-phospho (active) β-catenin, cyclin D1 and c-Myc [26]. Furthermore, circ-PGC silencing also induced cell apoptosis and suppressed the protein levels of MMP9, c-Myc, and β-catenin [158].

Based on studies, the PI3K/Akt signaling pathway in NSCLC cells could be stimulated by circ_0000376, circRNA_100876, circPVT1, circ_0016760, circ_0102231, hsa_circ_0018818 and restrained by circLIFR and circ-PLCD1 ceRNA networks that are discussed below [[165], [166], [167], [168], [169], [170], [171], [172]].

Circ_0000376, circRNA_100876, circ_0016760, circ_0102231 and hsa_circ_0018818 sequester miR-488-3p, miR-636, miR-646, miR-635 and miR-767-3p to upregulate BRD4, RET, AKT3, NOVA2 and Nidogen 1 expression, respectively and finally trigger PI3K/AKT signaling pathway. Downregulation of these circRNAs was indicated to induce apoptosis by releasing related miRNAs [[165], [166], [167], [168], [169]].

Circ_0000376 silencing was shown to induce PTEN protein [165]. In NSCLC tissues, circ_0102231 expression level was associated with the clinical stage [168].

Following radiation therapy, NSCLC cells had an increase in circPVT1, which acts as a ceRNA of miR-1208. CircPVT1 downregulation hampered PI3K/AKT/mTOR pathway by overexpressing miR-1208 [170].

On the other hand, circLIFR was reduced in NSCLC tissues and cells. CircLIFR upregulation suppressed AKT/PTEN signaling pathway via sponging miR-429, which could target CELF2 [171].

In NSCLC tissues and cell lines, circ-PLCD1 was suppressed and might be induced by protein p53 to facilitate apoptosis. Circ-PLCD1 elevation could induce PTEN expression to inhibit PI3K/AKT signaling through sponging miR-375 and miR-1179 [172].

ERK/JNK signaling pathway was inhibited in cells overexpressing hsa_circ_0004050, which was expressed at lower levels in tumor tissues from NSCLC patients. hsa_circ_0004050 could promote apoptosis via miR-1233-3p/DUSP9 axis [173].

The expression of BRD4 and MAPK‐activating death domain‐containing protein (MADD) in NSCLC cell lines was regulated via circ_0074027/miR‐185‐3p axis [174].

Other investigations revealed that in NSCLC, hsa_circ_0007580 and circ-RAD23B were upregulated and modulated PRKCA and MAP4K3 expression by sponging miR-545-3p and miR-142-3p, respectively. These circRNAs were shown to be associated with p38/MAPK signaling pathway activation [175,176]. Suppression of circ-RAD23B resulted in lower ERK1/2, JNK, and p38 phosphorylation, three fundamental molecules of the MAPK signaling pathway [176].

CircDENND2A sponges miR-34a to upregulate CCNE1 expression in NSCLC and prevent apoptosis. According to bioinformatic analysis, circDENND2A can affect TP53 signaling and the cell cycle [177].

In gefitinib-resistant NSCLC cells, circ_0091537 was overexpressed and sequestered miR-520h to induce YAP1 expression [178].

In NSCLC tissues, hsa_circ_0002874 had markedly higher levels and downregulated miR1273f to promote MDM2 expression. MDM2 inhibits P53 expression to modulate the cell cycle [179].

Hsa_circ_0014130 overexpression in NSCLC tissues induced the expression of Bcl-2 relatively by sponging miR136-5p [180].

Overexpression of circ-MEMO1 suppressed apoptosis by downregulating miR-101-3p, and promoting KRAS mRNA expression and glycolytic metabolism [181].

Lung adenocarcinoma (LUAD) is classified as a subtype of NSCLC [182]. In LUAD tissues and cells, circFOXP1 was significantly overexpressed and induced WNT1 upregulation by sponging miR-185-5p. WNT1 is an oncogene that has been associated with a number of developmental processes [183,184].

Yuanshan Yao revealed that in LUAD tissues and cells, circ_0018414 had low expression and could act as a sponge of miR-6807-3p to modulate DKK1 expression. DKK1 is an inhibitor of the Wnt/β-catenin pathway and can attach to LRP5/6. Induction of circ_0018414 expression reduced cancer progression and increased cell apoptosis by augmenting DKK1 expression to inhibit Wnt/β-catenin pathway activation [161].

Hippo pathway in LUAD cells was identified to be activated by hsa_circ_0017109 upregulation, which sponges miR-135b-3p to induce TOX3 expression. Additionally, it was proposed that melatonin might prevent the progression of LUAD [185].

#### 2.5.2 Breast cancer (BC)

Nowadays, breast cancer (BC) has become the most prevalent cancer in the world. The incidence of BC is closely related with human development [186].

In breast cancer, circNINL and circ_0008784 upregulation could indirectly affect Wnt/β-catenin activation through sponging miR-921 and miR-506–3p to promote ADAM9 and CTNNB1 (catenin beta 1) expression, respectively [187,188].

Previous investigations revealed that ADAM9 triggers β-catenin signaling, which induces the transcription of β-catenin signaling downstream genes such as CCND1, MMP9, and Survivin in BC. Survivin is a newly discovered member of the apoptosis-inhibitory protein family [187].

CircFAT1 decreased apoptosis in oxaliplatin-resistant BC cells by sponging miR-525-5p to regulate SKA1 expression. A positive correlation existed between the Notch and Wnt signaling pathways and SKA1 expression. Silencing SKA1 downregulated Notch2, GSK-3β, and β-catenin levels, implying the inhibition of the Notch and Wnt signaling pathways [189].

In contrast, circRNA-000911 acts as a tumor suppressor in BC. Upregulation of circRNA-000911 sponges miR-449a to induce the expression of Notch1. It was also identified that one of the most strongly suppressed pathways following circRNA-000911 overexpression was NF-κB signaling. The functional linkages between NF-κB signaling pathway and Notch1 are well acknowledged. NF-κB signaling was suppressed by the upregulation of circRNA-000911 [190].

Circ_0006528 upregulation induced the expression of Raf1 by sponging miR-7-5p and consequently triggering the MAPK/ERK pathway. Raf, MEK, and ERK mediate critical biological processes, such as cell proliferation, apoptosis, and metastasis [191].

AKT3 expression in BC was modulated by circWHSC1 via sponging miR-212-5p. CircWHSC1 suppression decreased TNBC progression and induced apoptosis [192].

#### 2.5.3 Glioma

Gliomas are referred to tumors arising from glial cells within the brain or spinal cord. Glioblastoma is a malignant and severe form of glioma, classified as grade IV according to the World Health Organization (WHO) classification system [193].

Glioma progression is induced by upregulating several circRNAs, such as circumHIPK3, circ-0014359, circNFIX, and circ_PTN, which ultimately activate different signaling pathways and regulate apoptosis [[194], [195], [196], [197]].

PI3K/AKT signaling pathway is induced by circHIPK3/miR-524-5p/KIF2A axis in TMZ-resistant glioma cells [194]. Circ-0014359 also promotes glioma progression by targeting miR-153/PI3K signaling pathway [195]. Notch signaling pathway is promoted by circNFIX/miR-34a-5p/NOTCH1 axis [196]. The circ_PTN/miR-122/SOX6 axis is considered to be regulated by the MAPK/ERK pathway [197].

Studies on glioblastoma have indicated that circABCC3, circ‐AHCY and circSERPINE2 are overexpressed in glioblastoma tissues and cells and sponge miR-770-5p, miR-1294 and miR-361-3p/miR-324-5p, respectively [[198], [199], [200]].

CircABCC3 indirectly targets SOX2 expression. CircABCC3 knockdown hampered PI3K/AKT pathway activation, while promoted cell apoptosis in glioblastoma [198].

Circ-AHCY triggers Wnt/β-catenin signaling pathway to modulate MYC expression, which induces CTNNB1 transcription. Circ-AHCY designates EIF4A3 to stabilize TCF4 mRNA. Increased levels of both β-catenin and TCF4 facilitate the stability of the TCF4/β-catenin complex. In turn, TCF4/β‐catenin activates circ‐AHCY transcription [199].

CircSERPINE2 sequesters miR-361-3p and miR-324-5p to control Bcl-2 expression to suppress the apoptosis of glioblastoma cells [200].

#### 2.5.4 Osteosarcoma (OS)

Osteosarcoma (OS) develops from mesenchymal cells and is characterized by rapid infiltrating development, early lung metastasis, and a high recurrence rate [201].

Two circRNAs are considered to be associated with Wnt/β-catenin pathway. Hsa_circ_0002052, which sponges miR-1205, had lower expression in OS tissues and cell lines, and its upregulation inactivated Wnt/β-catenin pathway by increasing APC2 expression. Therefore, APC2 negatively regulates the Wnt/β-catenin pathway [202].

On the other hand, circ_0003732 had higher expression in OS tissues and cells. Circ_0003732 modulates CPEB1 expression by sponging miR-377-3p and can trigger the Wnt/β-catenin signaling pathway [203].

Circ_001422 expression in OS progression was associated with advanced clinical stage, tumor size, and distant metastasis. Circ_001422 sequesters miR-195-5p to increase FGF2 expression. Additionally, this occurrence activates the PI3K/Akt pathway, which speeds up the advancement of OS through mechanisms such as apoptosis suppression [201].

Circ-0001785 was upregulated in OS cells and induced HOXB2 expression by sponging miR-1200. Circ-0001785 could influence OS cell apoptosis by controlling the Bcl-2 family and blocking the PI3K/Akt/mTOR pathway.

Knocking down circ-0001785 was demonstrated to affect antiapoptotic genes in Bcl-2 family and the proapoptotic gene Bad. Low expression of HOXB2 triggered caspase-9 and eventually increased apoptosis in OS cells [204].

The results of Deng's study suggested that circ_0009910 acts as a sponge of miR-449a to increase IL6R in OS. IL6R eventually triggered JAK1/STAT3 signaling. Additionally, upregulation of miR-449a suppressed p-JAK1, p-STAT3, cyclin D1, and Bcl-2 protein levels and promoted the expression of Bax [205].

Hsa_circ_0008035, which sponges miR-375, was overexpressed in OS serum, cells, and tissues. The Notch signaling pathway in OS cells was disrupted by downregulating hsa_circ_0008035, whereas apoptosis was induced [206].

Circ_0000376 induced the expression of the Bcl-2 protein by sequestering miR-432-5p in OS cells. Tan I (Tanshinone I) inhibited tumor growth by reversing the circ_0000376/miR-432-5p/Bcl-2 axis to trigger apoptosis in OS cells [207].

Circ_0004674 sponges miR-142-5p to upregulate Mcl-1, which is a member of the Bcl-2 family, to induce OS progression and chemoresistance [208].

#### 2.5.5 Thyroid cancer

Women are three times more likely to contract thyroid cancer than men, resulting in a disparity by sex.

In thyroid cancer cells, circNEK6 was overexpressed, triggering the Wnt signaling pathway and inhibiting apoptosis by sponging miR-370-3p to promote FZD8 expression [209].

Circ-ITCH was downregulated in papillary thyroid cancer (PTC) tissues and cells. Circ-ITCH overexpression acts as a tumor suppressor and prevents PTC progression by sequestering miR-22-3p. Circ-ITCH increases CBL (an E3 ligase of nuclear β-catenin) expression to hamper Wnt/β-catenin pathway activation [210].

PI3K/Akt signaling is activated by circ_PSD3. This circRNA enhances PTC development by hindering apoptosis through the miR-637/HEMGN axis [211].

Circ_0079558 upregulation sponges miR-26b-5p to increase MET expression and eventually MET/AKT signaling activation in PTC. Previous studies revealed that there is generally an association between MET and EGFR and that EGFR overexpression increases AKT phosphorylation [212].

#### 2.5.6 Nonfunctioning pituitary adenomas (NFPAs)

Nonfunctioning pituitary adenomas (NFPAs) are the major cause of hypopituitarism and infertility. CircOMA1 enhanced NFPA development by sponging miR-145-5p to induce TPT1 signaling pathway. MiR-145-5p upregulation, as a tumor suppressor, repressed TPT1 and its downstream factors Mcl-1 and Bcl-xL, whereas increased Bax [213].

#### 2.5.7 Laryngeal cancer (LC)

Approximately 50 % of head and neck tumors are laryngeal carcinomas (LCs). In both LC tissues and cells, the expression level of circ_PVT1 was increased, and CBX4 expression was promoted by sponging miR-21-5p, which ultimately activated Wnt4/β-catenin signaling pathway. Circ_PVT1 silencing suppressed Bcl-2, p53, and p21 expression and induced cyclin-D1, Bax, and cleaved caspases 3 and 9 expression in LC cells [214].

#### 2.5.8 Ameloblastoma

Ameloblastoma, as the most prevalent odontogenic epithelial tumor, has a high probability of postoperative recurrence and a locally aggressive predisposition. Hsa_circ_0089153 overexpression in ameloblastoma sponges hsa-miR-608 to regulate EGFR p53 and eventually activate the MAPK signaling pathway during ameloblastoma progression [215].

The ceRNA networks of other cancers are listed in Table 5.

**Table 5:** Other cancers.

CircRNA	Expression	miRNA	Target mRNA	Signaling pathway	Ref
**Lung cancer**					
circHIPK3	up-regulated	miR-381-3p	–	Akt/mTOR	[25]
circ_0000190	up-regulated	miR-130a-3p	Notch-1	Notch	[162]
circVAPA	up-regulated	miR-377-3p, miR-494-3p	IGF1R	PI3K/Akt	[163]
circ_0017109	up-regulated	miR-671-5p	FZD4	Wnt/β-catenin	[26]
circ-PGC	up-regulated	miR-532-3p	FOXR2	Wnt/β-catenin	[158]
circ-ZNF124	up-regulated	miR-498	YES1	Wnt/β-catenin	[164]
circ_0000376	up-regulated	miR-488-3p	BRD4	PI3K/PKB	[165]
circRNA_100876	up-regulated	miR-636	RET	PI3K/Akt	[166]
circ-PLCD1	down-regulated	miR-375, miR-1179	PTEN	PI3K/Akt	[172]
circPVT1	up-regulated	miR-1208	–	PI3K/Akt/mTOR	[170]
circLIFR	down-regulated	miR-429	CELF2	PTEN/Akt	[171]
circ_0102231	up-regulated	miR-635	NOVA2	PI3K/Akt	[168]
hsa_circ_0018818	up-regulated	miR-767-3p	NID1	PI3K/Akt	[169]
circ_0016760	up-regulated	miR-646	AKT3	PI3K/Akt	[167]
hsa_circ_0004050	down-regulated	miR-1233-3p	DUSP9	ERK/JNK	[173]
circ_0074027	up-regulated	miR‐185‐3p	BRD4 MADD	MAPK	[174]
hsa_circ_0007580	up-regulated	miR-545-3p	PRKCA	P38/MAPK	[175]
circ-RAD23B	up-regulated	miR-142-3p	MAP4K3	MAPK	[176]
circDENND2A	up-regulated	miR-34a	CCNE1	p53	[177]
circ_0091537	up-regulated	miR-520h	YAP1	YAP1	[178]
hsa_circ_0002874	up-regulated	miR1273f	MDM2	P53	[179]
hsa_circ_0014130	up-regulated	miR136-5p	Bcl-2	Bcl-2	[180]
circ-MEMO1	up-regulated	miR-101-3p	KRAS	RAS/MAPK	[181]
circFOXP1	up-regulated	miR-185-5p	WNT1	Wnt	[183]
circ_0018414	down-regulated	miR-6807-3p	DKK1	Wnt/β-catenin	[161]
hsa_circ_0017109	up-regulated	miR-135b-3p	TOX3	Hippo	[185]
**Breast cancer**					
circNINL	up-regulated	miR-921	ADAM9	β-catenin	[187]
circ_0008784	up-regulated	miR-506–3p	CTNNB1	Wnt/β-catenin	[188]
circFAT1	up-regulated	miR-525- 5p	SKA1	Notch & Wnt	[189]
circRNA-000911	down-regulated	miR-449a	Notch1	Notch & NF-κB	[190]
circ_0006528	up-regulated	miR-7-5p	Raf1	MAPK/ERK	[191]
circWHSC1	up-regulated	miR-212-5p	AKT3	Akt	[192]
**Glioma**					
circABCC3	up-regulated	miR-770-5p	SOX2	PI3K/Akt	[198]
circHIPK3	up-regulated	miR-524-5p	KIF2A	PI3K/Akt	[194]
circ-0014359	up-regulated	miR-153	–	PI3K	[195]
circ‐AHCY	up-regulated	miR-1294	MYC	Wnt/β-catenin	[199]
circNFIX	up-regulated	miR-34a-5p	Notch1	Notch	[196]
circ_PTN	up-regulated	miR-122	SOX6	MAPK/ERK	[197]
circSERPINE2	up-regulated	miR-361-3p, miR-324-5p	Bcl-2	–	[200]
**Osteosarcoma**					
hsa_circ_0002052	down-regulated	miR-1205	APC2	Wnt/β-catenin	[202]
circ_0003732	up-regulated	miR-377-3p	CPEB1	Wnt/β-catenin	[203]
circ_001422	up-regulated	miR-195-5p	FGF2	PI3K/Akt	[201]
circ-0001785	up-regulated	miR-1200	HOXB2	PI3K/Akt/mTOR	[204]
circ_0009910	up-regulated	miR-449a	IL6R	JAK1/STAT3	[205]
hsa_circ_0008035	up-regulated	miR-375	–	Notch	[206]
circ_0000376	up-regulated	miR-432-5p	Bcl-2	–	[207]
circ_0004674	up-regulated	miR-142-5p	Mcl-1	–	[208]
**Thyroid cancer**					
circNEK6	up-regulated	miR-370-3p	FZD8	Wnt	[209]
circ-ITCH	down-regulated	mir-22-3p	CBL	Wnt/β-catenin	[210]
circ_PSD3	up-regulated	miR-637	HEMGN	PI3K/Akt	[211]
circ_0079558	up-regulated	miR-26b-5p	MET	MET/Akt	[212]
**Nonfunctioning pituitary adenomas (NFPAs)**					
circOMA1	up-regulated	miR-145-5p	TPT1	TPT1/Mcl-1/Bcl-xL/Bax	[213]
**Laryngeal cancer**					
circ_PVT1	up-regulated	miR-21-5p	CBX4	Wnt/β-catenin	[214]
**Ameloblastoma**					
hsa_circ_0089153	up-regulated	miR-608	EGFR p53	MAPK	[215]

## 3 Conclusion and future perspective

Cell homeostasis is dependent upon cell signaling pathways, and aberrations in the regular state of these signaling pathways could lead to various complications, especially cancer development [216]. Tumor progression might result from dysregulation between cell cycle and apoptosis; based on multiple recent studies, this balance could undergo epigenetic alterations via ceRNA networks [217].

Bioinformatics tools have facilitated ceRNA network research; however, experimental verification is necessary for data confirmation [218]. Hsa_circ_0085494–hsa-miR-330-3p–TGFBR3 axis is predicted to be involved in prostate cancer [219]. Bioinformatic analysis of Jiao Lyu suggested TET1-hsa_circ_0093996-miR183-PDCD4 as a potential regulatory axis in the pathogenesis of retinoblastoma [220].

Identifying diagnostic biomarkers and therapeutic targets in cancer has been a challenge for researchers. An ideal diagnostic biomarker should have stable expression in body fluids such as serum and plasma and could be easily detected by available diagnostic methods [221]. Circ_0006528 overexpression was revealed to be notably associated with advanced tumor-node-metastasis (TNM) stage and poor prognosis in patients with breast cancer [191]. High circ_0067997 expression was associated with a poor overall survival rate in gastric cancer patients [121].

However, most of these studies are based on tissue and cell line research. On the other hand, therapeutic biomarkers should be evaluated through multifactorial *in vivo* investigations to consider the complex interplay between different physiological processes that may impact cell signaling, including immune responses and paracrine activities [222].

Since cell signaling-associated ceRNA networks have mostly exhibited cancer-specific abnormal expressions, they are considered potential therapeutic biomarkers.

CircRNA-MYLK has been identified to potentially function as a ceRNA in both bladder and cervical cancer tissues; nevertheless, this circRNA acts by sponging dissimilar miRNAs, thus leading to distinct signaling pathways [130,140]. Therefore, nonspecific circRNAs, which regulate different signaling pathways in various carcinomas, may not be ideal targets for the treatment of specific cancers.

Hao Zheng et al. [162] suggested luteolin as a probable therapeutic target for lung cancer, which inhibited lung cancer progression and Notch-1 signaling pathway and promoted apoptosis by regulating the circ_0000190/miR-130a-3p axis *in vitro*. Aloperine has been shown to suppress the development of colorectal cancer cells and induce apoptosis by modulating the circNSUN2/miR-296-5p/STAT3 pathway. Moreover, Aloperine may increase the aberrantly low expression of miR-296-5p in colorectal cancer [112].

CeRNA network may also have an impact on chemoresistance in certain cancers; for instance, it has been shown that silencing circ_0004674 inhibited osteosarcoma cell resistance to doxorubicin and promoted cell cycle arrest and apoptosis *in vitro* and *in vivo* via miR-142-5p/Mcl-1 axis [208].

Targeting apoptosis in tumor tissues has been one of the main approaches in clinical cancer therapy. A therapeutic perspective from the aspect of apoptosis involves modulating apoptotic molecules and related signaling pathways through ceRNA networks, particularly those targeting various networks in a specific type of cancer simultaneously. Mechanistically, we can induce tumor suppressor molecules or repress oncogene molecules in targeted networks to inhibit cancer progression. The major goal of this review is to consider ceRNA networks as targets for agents that can stably control the expression of circRNAs to regulate apoptosis through cell signaling pathways in cancers.

However, more research is required to determine the precise regulatory mechanisms of these networks and tumor progression. Besides, additional investigations are needed to confirm whether the targeted therapeutic pathway would operate in a tissue-specific way.

## Funding

No funding.

## CRediT authorship contribution statement

**Mina Shahpari:** Data curation, Investigation, Project administration, Resources, Writing – original draft, Writing – review & editing. **MohamadReza Hashemi:** Visualization, Writing – original draft, Writing – review & editing, Project administration. **Tayebeh Younesirad:** Resources, Writing – original draft. **Aida Hasanzadeh:** Writing – original draft. **Mohammad mahdi Mosanne:** Writing – original draft. **Mohamadreza Ahmadifard:** Conceptualization, Investigation, Supervision.

## Declaration of competing interest

The authors declare that they have no known competing financial interests or personal relationships that could have appeared to influence the work reported in this paper.
